# Quantification of HER2 heterogeneity in breast cancer–implications for identification of sub-dominant clones for personalised treatment

**DOI:** 10.1038/srep23383

**Published:** 2016-03-21

**Authors:** Niamh E. Buckley, Claire Forde, Darragh G. McArt, David P. Boyle, Paul B. Mullan, Jacqueline A. James, Perry Maxwell, Stephen McQuaid, Manuel Salto-Tellez

**Affiliations:** 1 Centre for Cancer Research and Cell Biology, Queen’s University, Belfast, UK; 2 Tissue Pathology, Belfast Health and Social Care Trust, Belfast City Hospital, Lisburn Road., Belfast, UK.

## Abstract

Breast cancer is a heterogeneous disease, at both an inter- and intra-tumoural level. Appreciating heterogeneity through the application of biomarkers and molecular signatures adds complexity to tumour taxonomy but is key to personalising diagnosis, treatment and prognosis. The extent to which heterogeneity exists, and its interpretation remains a challenge to pathologists. Using HER2 as an exemplar, we have developed a simple reproducible heterogeneity index. Cell-to-cell HER2 heterogeneity was extensive in a proportion of both reported ‘amplified’ and ‘non-amplified’ cases. The highest levels of heterogeneity objectively identified occurred in borderline categories and higher ratio non-amplified cases. A case with particularly striking heterogeneity was analysed further with an array of biomarkers in order to assign a molecular diagnosis. Broad biological complexity was evident. In essence, interpretation, depending on the area of tumour sampled, could have been one of three distinct phenotypes, each of which would infer different therapeutic interventions. Therefore, we recommend that heterogeneity is assessed and taken into account when determining treatment options.

Personalised medicine centers on the paradigm of inter-tumoural heterogeneity, by recognizing that each patient’s tumour is a unique disease entity with a definable molecular fingerprint. Increasingly, this concept of uniqueness is being extended to further incorporate those genotypic differences observed within regions of individual tumours and associated metastatic deposits, referred to as intra-tumoural heterogeneity^1^.

Intra-tumoural heterogeneity in breast cancer has been acknowledged for some time. Its existence may partially explain why breast cancer remains a challenging disease to treat, with significant morbidity and mortality, despite the use of well-established targeted therapies. In the UK, breast cancer is the third most common cause of cancer death, and is accountable for 7% of all cancer-related mortality^2^. Quantifying the extent of intra-tumoural heterogeneity may improve both predictive and prognostic biomarker assays.

One such biomarker with reported heterogeneity in breast cancer is the Human Epidermal Growth Factor Receptor 2 (HER2), a member of the EGF receptor (EGFR) family. For invasive breast cancers that overexpress HER2 protein (reported range between 15–30%)^3^, trastuzumab offers a highly effective targeted therapy that can ameliorate the prognostic deficit inferred on patients with HER2 gene amplification. Treatment response rates, however, are variable. Optimal treatment requires that assay timings and techniques provide an accurate and true summary of an individual patient’s HER2 status. However, heterogeneous amplification of the HER2 gene is associated with cancer progression and reduced disease free survival^4^. Examples of discordant HER2 assay results between core biopsy material and resection specimens have been documented. Though, at least one meta-analysis (with data from 646 tumours) showed overall concordance of 97.8%^5^,^6^. If HER2 assessment in cores is generally representative of resections then does assessment of heterogeneity show any major differences between these two sample types?

Therefore, we sought to address two issues in this study. The first concerned reliable and reproducible quantification of HER2 heterogeneity in breast cancer. The second, whether HER2 heterogeneity is specific or a reflection of broader molecular heterogeneity.

We reviewed the extent of heterogeneity within tissue samples assessed for HER2 gene amplification in routine diagnostic practice within the Northern Ireland Molecular Pathology Laboratory (NIMPL). We retrospectively quantified heterogeneity within clinical samples using defined heterogeneity indices, correlated these with subjective assessments of cellular heterogeneity and compared heterogeneity across the different sampling methods of needle core biopsy (NCB) versus resection.

To address the second issue we assessed variation in molecular subtype in an index case with striking clonal heterogeneity. To illustrate this concept we analysed primary and metastatic lesions with an array of IHC biomarkers to classify molecular subtype, identify areas of diagnostic discrepancy, and infer therapeutic interventions.

## Results

**Figure 1:**
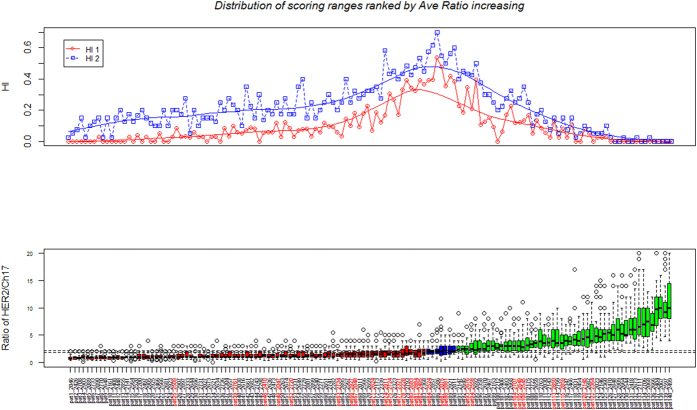
Composite image showing cases arranged according to increasing HER2/Chr17 ratios. The top graph shows the HER2/Chr17 ratio spread of individual cells per case. Non-amplified cases are shown red, borderline blue and amplified green. The bottom graph shows the H1 and H2 scores for the same samples. The samples names highlighted in red indicate cases previously assessed as showing heterogeneity by HER2 IHC.

A total of 140 cases were available for analysis. Of these, 83 had been clinically categorised as non-amplified (ratio <1.8), 50 as amplified (ratio >2.2), 5 as borderline amplified (ratio 2.0-to-2.2) and 2 as borderline non-amplified (ratio 1.8-to-2.0). The degree of cell HER2/Chr17 ratio dispersion per case across the entire cohort is presented in Fig. 1. Greater dispersion of ratio per case occurs generally as overall average ratio increases. However, HI (HI1 or HI2) defined heterogeneity is greatest in higher ratio non-amplified, borderline and lower ratio amplified cases. Highly amplified cases show the greatest ratio dispersion but are also defined by more uniform amplification. The lowest ratio non-amplified cases show very little ratio dispersion and uniform non-amplification.

### Non-amplified cases

Sixty-one (73%) cases showed heterogeneity according to HI1. However, all of the non-amplified cases reviewed showed cell-to-cell heterogeneity according to HI2. Forty-four cases (53%) showed 5–50% of tumour cells with a HER2/Chr17 ratio >2.2. Maximum average HER2/Chr17 ratios were higher for cases showing any heterogeneity versus those with uniformly non-amplified cells (1.74 vs 1.2); minimum ratios were similar (0.8 vs 0.77). By the HI2 index 77 (93%) cases demonstrated at least 5% but fewer than 50% cells that were amplified.

### Amplified cases

Thirty-seven (74%) cases showed heterogeneity according to HI1. Thirty-six cases had between 5–50% of tumour cells with a HER2/Chr17 ratio <1.8. Purely amplified cases (i.e.HI1 equivalent to 0) had higher maximum, minimum, mean and median HER2/Chr17 ratio compared to cases with any heterogeneity (10.45; 4.14; 6.68; 5.99 vs 7; 2.42; 3.62; 3.24). These cases had HER2/Chr17 ratios ranging from 4.14 to 10.45 compared to 2.42 to 7 for cases showing any heterogeneity. Forty-two (84%) cases showed heterogeneity according to HI2. The purely amplified cases had HER2/Chr17 ratios ranging from 5.2 to 10.5.

### Borderline cases

As expected, reported borderline cases (n = 7) displayed high levels of heterogeneity relative to the non-amplified and amplified groups. HI1 indices ranged from 0.23 to 0.6, HI2 from 0.45 to 0.8.

### Subjective versus objective heterogeneity

HER2 gene heterogeneity measurements are shown in Fig. 1 compared to subjective assessments of HER2 IHC heterogeneity as recorded during clinical assessment (cases highlighted in red). A total of 28 cases were previously assessed as showing heterogeneity. The majority of these cases clustered either side of ratios indeterminate HER2/Chr17 amplification. As shown, these tended to have higher HI1 and HI2 ratios.

### Specimen type

**Figure 2:**
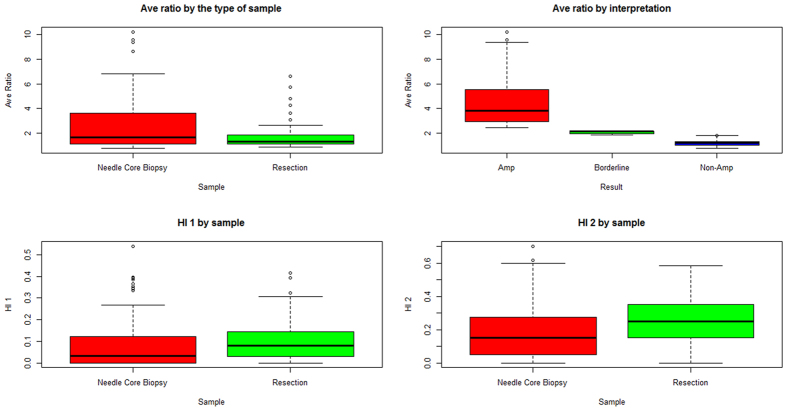
Box and Whisker plots of average Her2/Chr17 rations between Needle Core Biopsy and Resection or Amplified, Borderline or Non-Amplified Cases. Box and Whisker plot of HI1 or HI2 scores between Needle Core Biopsy and Resection.

Heterogeneity index values were compared according to sample type tested in order to determine if the nature of the specimen impacted on the degree of heterogeneity shown. After excluding equivocal cases and those where the nature of the sample was not recorded, HI1 and HI2 values were calculated depending on whether they were resection specimens or needle core biopsies (Fig. 2). No substantial differences in spread of data were identified between core and resection specimens.

### Case example

**Figure 3:**
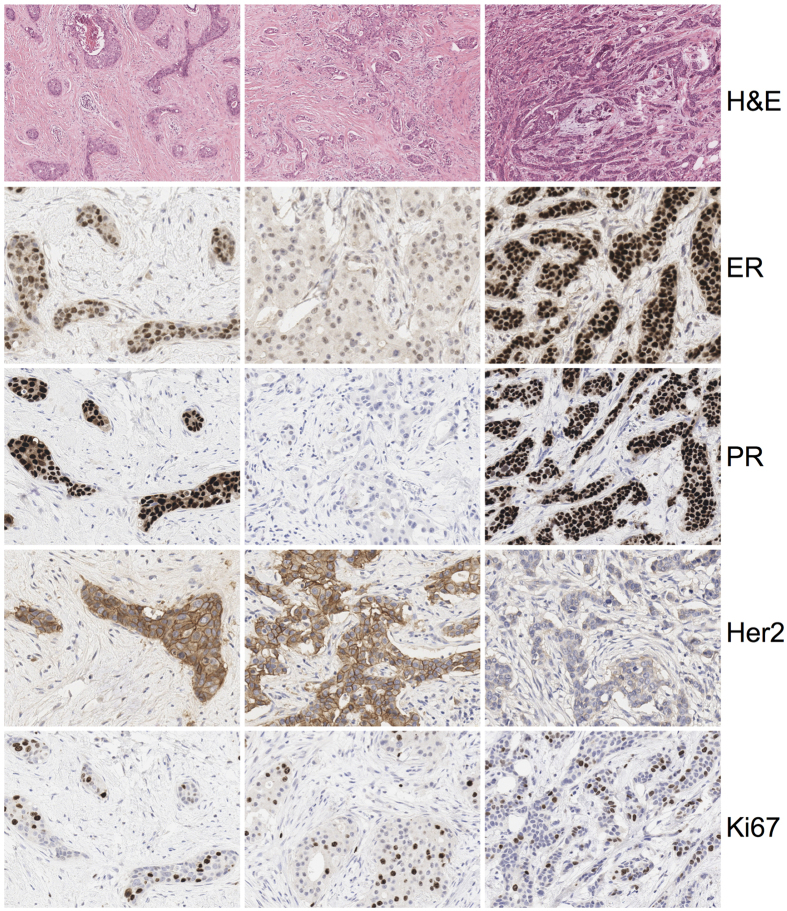
The 3 distinctly heterogeneous areas are shown in columns A to C with their biomarker profiles beneath for ER, PR, HER2 and Ki67.

Sections taken from the primary tumour displayed distinct well-segregated spatial morphological heterogeneity with H&E (Fig. 3). IHC heterogeneity largely conforming to these same areas was observed. Given that distinct clonal areas were readily identified, molecular subtypes could be assigned to adjacent areas in the primary tumour resection and lymph node metastasis.

The primary tumour contained areas with differing HER2 expression. Moreover, there was evidence of luminal B HER2 positive (LBHP), HER2 enriched (HE) and luminal A (LA) subgroups in separate areas (Fig. 3). The HER2 enriched area harboured intratumoural DCIS with a LBHP phenotype while the LA area contained focal DCIS with a HER2 enriched phenotype. A high degree of heterogeneity was also observed with the additional biomarkers p53 and p-mTOR, EGFR and IGF1R (Supp Figure 1).

**Figure 4:**
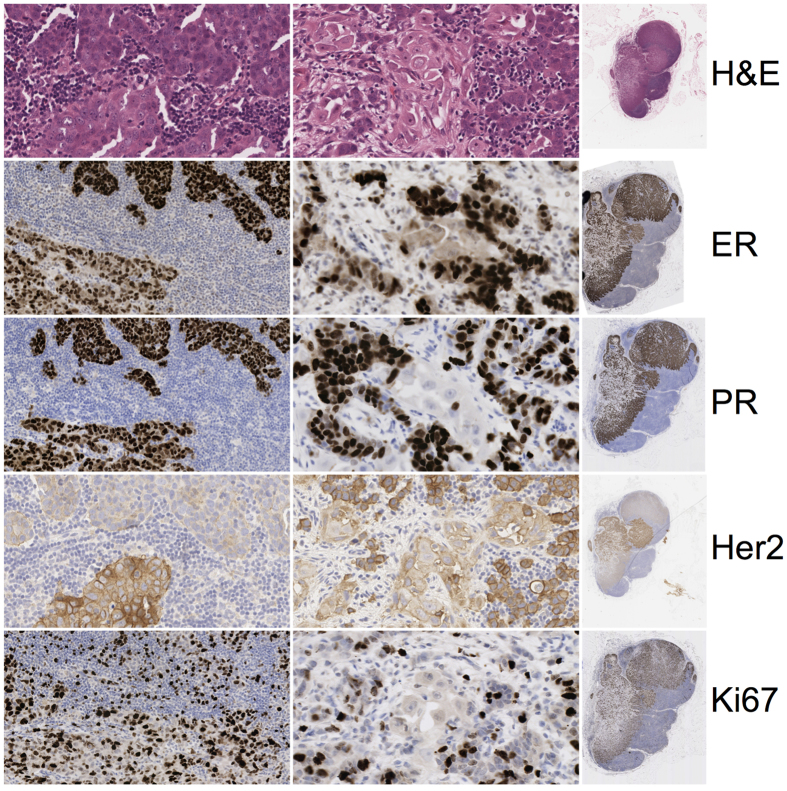
The metastatic deposit of tumour in the lymph node is shown. Overall lymph node biomarker expression of ER, PR, HER2 and Ki67 are shown for each stain in column C. The interface between 2 heterogeneous metastatic areas is shown in column A. A third more dispersed, though morphologically distinct population is shown in column B.

The lymph node contained 3 populations of metastatic cells also showing differing HER2 expression (Fig. 4). Two of these were well segregated. However, a third population of morphologically distinct malignant cells with a dispersed pattern was apparent. The lymph node therefore contained different molecular subtypes: LBHP, LBHN and triple negative basal phenotypes. Again varied expression of p53, p-mTOR, EGFR and IGF1R were noted (Supp Figure 2).

The luminal B HER2 positive group was present in both primary and nodal metastatic tumours. The HER2 enriched phenotype was detected in DCIS and primary tumour but not the nodal deposit. The node contained a triple negative basal phenotype deposit that was not detected in the primary resection specimen.

## Discussion

In this current study, using the proposed HI indices, we objectively scored and quantified heterogeneity in HER2 gene amplification. We identified a subset of patients with a sizable proportion of cells which amplification status differed to the overall assigned HER2 status. All non-amplified and most amplified cases showed heterogeneity according to the HI2 index, suggesting that this might be an overly sensitive measure. However, the HI1 index showed high ratio increases, corresponding to the greatest density of subjectively assessed heterogeneous cases. In the age of personalized medicine, the quantification of HER2 heterogeneity through the indices described may facilitate reliable heterogeneity assessment in routine practice to aid prediction, prognostication, and future research. As illustrated by the index case, there are implications of HER2 heterogeneity more widely as an indicator of underlying different molecular phenotypes.

The question invariably asked is if a higher degree of cell-to-cell heterogeneity impacts upon the clinical outcomes of patients? Unfortunately clinical follow-up is limited in the patient cohort. It is intuitive, however, that the proportion of identifiable subdominant clones within a patient’s tissue sample may impact on treatment response and prognosis^7^. Indeed, a recent publication indicates a significant association with reduced disease-free survival^4^. Elegant *in vivo* models^8^,^9^,^10^,^11^ have demonstrated the importance of subdominant clones within tumours in providing key paracrine signals to influence multiple aspects of tumour biology. The nature of these tumour/subclone interactions are complex and far from being fully elucidated^12^. However, targeting these minor clones may be an alternate or even complimentary approach if a favorable dominant tumour-promoting microenvironment is indeed reliant on signals from subclones.

Non-amplified cases with a high heterogeneity index, could be candidates for retesting if recurrence or progression occurs as, theoretically, under-recognised previously non-dominant HER2 positive cells become the majority. Determining HER2 heterogeneity in non-amplified breast cores may also indicate regional heterogeneity that would necessitate retesting in resection specimens^4^. HER2 heterogeneity has been assessed in a number of studies using a range of techniques such as IHC, and FISH. This is comprehensibly reviewed in Potts *et al.*^13^. In this study the authors develop a measure of HER2 IHC heterogeneity. Our study therefore represent a natural progression of this and we also note similar findings such as highest heterogeneity in cases initially identified as 2+ by IHC. However, we did not note high HI1 scores in cases where the HER2/Chr17 ratio was discordant between assessors. There are counter-arguments that HER2 heterogeneity has little prognostic significance. Patients with highly amplified tumours respond better to anthracycline therapy^14^ and apparent cell-to-cell heterogeneity may be an artefactual phenomenon^15^. In the HERA trial all patients received anthracycline therapy and those with borderline and amplified HER2 tumours had no significant differences in prognosis^14^. This may be due to interactions with targets of anthracyclines, such as TOP2a, that reside in close physical proximity to HER2 on the 17q12-q21 amplicon.

The detailed analysis of our index case reflects the broader molecular heterogeneity, beyond HER2, that may exist within certain breast cancers and metastases. Although an extreme example, it illustrates over the potential shortcomings of the methods used to classify molecular subtypes in breast cancer. The varying molecular profile observed would have consequences for therapy offered (trastuzimab, tamoxifen or neither), as well as outcomes in novel trials. Although most breast tumours do not display such extreme and clear-cut heterogeneous areas, individual tumour variation may occur at an undetectable level using current investigative approaches. The question concerning whether predictive and prognostic biomarkers should be tested on samples beyond the primary tumour is pertinent as exemplified by the differences between the molecular profiles of the primary and metastatic components of this case.

## Conclusion

Researchers must be cognizant of biomarker variance in breast cancer specimens when transferring initial laboratory based results to clinical samples. Furthermore, ignoring the possibility of differential biomarker expression across lesional stages (differences in primary tumour and lymph node expression) negates a holistic approach to tumour characterisation and consequent treatment. In addition, these findings emphasize the possibility of erroneously basing treatment decisions on negative, or indeed positive, results obtained at core biopsy.

## Materials and Methods

Ethical approval was obtained and tissue acquired through the Northern Ireland Biobank (NIB ref: 12–00017) and all work was carried out in accordance with the approved guidelines. Informed consent was obtained for all samples. For the analysis of HER2 genomic heterogeneity, 140 consecutive cases were reviewed from the NIMPL archives. Basic clinical information for these cases is shown in Table 1. These had been referred for HER2 status determination between October 2012 and September 2013 based on pathological interpretation in 3–4 hospitals served by the NIMPL. All of these cases had HER2 dual-color dual-hapten brightfield *in situ* hybridization (DDISH) applied and scored in keeping with UK recommendations^16^. Briefly, FFPE sections were stained with a HER2/CHR17 probe cocktail (Ventana cat no. 800 4422) as previously described^17^. Ninety-three of these were needle core biopsies, the remainder comprising larger resections. Manual enumeration of the HER2 and chromosome 17 signals was performed in a Clinical Pathological Accreditation (CPA) laboratory within areas of invasive tumour by two scorers (pathologist and clinical scientist) who analysed a total of 40 call nuclei in general (13 cases required additional assessment of 20 nuclei and 4 cases required an additional 40 nuclei to be assessed when discordant results impacted on clinical management). Scoring areas were selected on the basis of the IHC expression and focused on the focal 2+ regions within each sample. The case with striking heterogeneity was identified during routine reporting.

### Heterogeneity Index Calculation

The College of American Pathologists (CAP) published a recommendation in 2009, as an extension of their 2007 scoring guidelines, that defines HER2 genetic heterogeneity as the presence of tumour cells with HER2/chromosome 17 (HER2/Chr17) signal ratios of >2.2 in 5–50% of tumour cells analysed in an otherwise non-amplified sample. If >50% cells had a ratio >2.2, this was termed amplified^7^. We modified this approach to measure heterogeneous cell populations in both non-amplified and amplified specimens: the proportion of cells within predominantly amplified samples that had HER2/Chr17 ratios <1.8 or the proportion of cells within predominantly non-amplified samples that had HER2/Chr17 ratios >2.2. We also applied this calculation to a small number of cases with equivocal HER2 status.

For each sample scored in NI-MPL within the defined time period, all of the archived DDISH HER2 counting tables used for diagnostic purposes were reviewed. Available data included the absolute numbers of HER2 and Chr17 signals per cell used in the analysis. Using these data, 2 different indices were calculated for quantification of heterogeneity.

#### Heterogeneity Index 1 (HI1)

For cases originally reported as non-amplified, the number of amplified cells was assessed as a measure of heterogeneity. However, cells with borderline values were excluded (ratios 1.8–2.2 inclusive). In each case, the sum of the cells with individual amplified status (based on HER2/Chr17 ratio >2.2) was divided by the total number of cells counted. This calculation provided the HI1 value. A similar principle was applied to originally reported amplified cases wherein the sum of individually non-amplified cells (based on HER2/Chr17 ratio <1.8) was divided by the total number of cells counted.

#### Heterogeneity Index 2 (HI2)

While using a similar approach to HI1, HI2 also considered individual cells with borderline status. For cases originally reported as non-amplified, borderline cells were considered together with amplified cells. Conversely, for cases originally reported as amplified, borderline cells were considered together with non-amplified cells.

**Figure 5:**
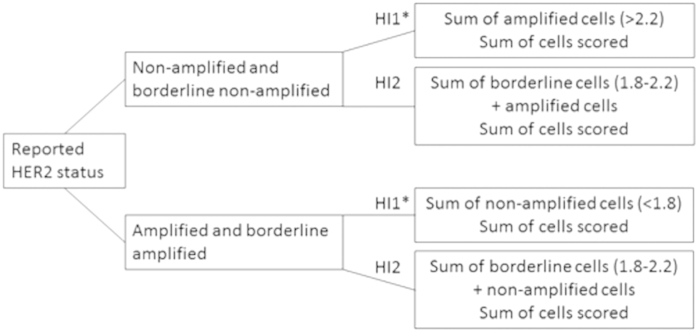
Summary of methods used to derive HI1 and HI2.

Six cases originally classified as borderline were assessed in a similar manner as above depending on whether they were borderline amplified or borderline non-amplified. The approach to assigning heterogeneity indices is summarized in Fig. 5. The degree of data spread was plotted using the Tukey box plot method.

During routine assessment, it is standard practice within our institution to note subjectively the presence of heterogeneity. Heterogeneity indices were compared with cases noted subjectively to have displayed heterogeneity.

### Immunohistochemistry

Additional IHC (ER, PR, HER2, Ki-67, p53, EGFR, p-mTOR and IGF1R) was performed on the case with striking heterogeneity. Sections for IHC were cut at 4 microns on a rotary microtome and dried at 37 °C overnight. All IHC was performed on automated immunostainers (Ventana Discovery or Leica BondMaX). Validated and optimised protocols were selected for each biomarker (see Table 2). Antigen binding sites were detected with Omni anti-rabbit or mouse detection system (Ventana cat no. 760–4310 or 760–4311) or a polymer based detection system (Bond cat no. DS 9800). All sections were visualized with DAB, counterstained in haematoxylin and mounted in DPX.

All IHC was interpreted by 2 pathologists (DB and MST). When disagreement on interpretation occurred, cases were reviewed by both pathologists on a multihead microscope to reach a consensus opinion. ER and PR were assessed using the quick score method which considers proportion and intensity of nuclear expression: scores ≤3/8 were considered positive (i.e. ≤1% cells expressing ER)^18^. HER2 analysis considered membranous expression and was based on the Bond Oracle Test method: scores of 3+ or 2+ with confirmatory ISH were considered positive^19^. As described elsewhere, p53 interpretation followed a 3 tier system where complete absence and confluent strong positivity were considered aberrant while all intermediary expression was considered non-aberrant^17^.

EGFR analysis considered membranous expression only. In keeping with the description of interpretation by Chaeng *et al.*, any definite expression was considered as positive^20^. Ki67 nuclear expression was used to ascertain Ki67 index: a threshold of 14% expression was used to distinguish low and high proliferation indices^21^. IGF1R membranous expression was almost ubiquitous across all slides and cases and therefore only intensity (weak, moderate, strong) was considered in interpretation. Mild cytoplasmic p-mTOR expression in >5% of malignant cells was considered positive.

### Molecular subtype classification of breast cancers using surrogate IHC

The IHC markers (ER, PR, HER2 and Ki67) were used to classify tumour areas as luminal A (LA: ER^+^, PgR^+^, HER2^−^, Ki67 low), luminal B HER2 negative (LBHN: ER^+^, HER2^−^ and at least one of Ki67 high or PgR^−^), luminal B HER2 positive (LBHP: ER^+^, HER2^+^), HER2 enriched (HE: HER2^+^, ER^−^ and PgR^−^) and triple-negative (TN: ER^−^, PgR^−^, HER2^−^)^21^.

We considered two types of heterogeneity in assessing the example case. The lesions of interest were primary invasive ductal carcinoma (IDC) and lymph node metastatic deposits (LNM). Distinctly separated differences in expression for a particular lesion type were termed spatial heterogeneity (SH). Differences between the lesion types were termed temporal heterogeneity (TH). The presence of heterogeneity was assessed for single biomarkers as well as panels conforming to molecular subtypes.

## Additional Information

**How to cite this article**: Buckley, N. E. *et al.* Quantification of HER2 heterogeneity in breast cancer – implications for identification of sub-dominant clones for personalised treatment. *Sci. Rep.* **6**, 23383; doi: 10.1038/srep23383 (2016).

## Supplementary Material

Supplementary Information

## Figures and Tables

**Table 1:** Summary of basic clinicopathological information for patient cohort.

Age	Range; Median	33–88; 59
Tcode	T1	51/121
	T2	48/121
	T3	13/121
	T4	9/121
Ncode	N0	57/121
	N1	35/121
	N2	13/121
	N3	16/121
Mcode	M0	109/117
	M1	8/117
Primary or Metastatic	Primary disease	121/125
	Metastatic disease	4/125
Subtype	IDC	66/139
	IDC + DCIS	58/139
	Mixed	5/139
	ILC	10/139

**Table 2:** Summary of antibody dilutions and detection.

Antibody	Clone	Dilution	Company	Automated platform
ER	6F11	1:200	Leica	Leica BOND-MAX
HER2	CB11	Pre-set dilution	Leica	Leica BOND-MAX
p53	DO-7	1:100	Dako	Leica BOND-MAX
PR	636	1:150	Dako	Leica BOND-MAX
Ki67	MM1	1:200	Leica	Leica BOND-MAX
EGFR	3C6	Pre-set dilution	Ventana	Ventana DISCOVERY XT
IGF1R	G11	Pre-set dilution	Ventana	Ventana DISCOVERY XT
p-mTOR	49F9	1:100	Cell Signalling	Leica BOND-MAX
