# Supplementary material for: Quantification of HER2 heterogeneity in breast cancer–implications for identification of sub-dominant clones for personalised treatment
Source: Sci Rep. 2016 Mar 21;6:23383. doi: 10.1038/srep23383 (PMC4800308; doi:10.1038/srep23383)
Supplement: Supplementary Information [file srep23383-s1.pdf]

**Quantification of HER2 heterogeneity in breast cancer – implications for identification of sub-dominant clones for personalised treatment.**

Niamh E Buckley[1], Claire Forde[1], Darragh G McArt[1], David P. Boyle[1,2], Paul B. Mullan[1], Jacqueline A James[1, 2], Perry Maxwell[1, 2], Stephen McQuaid[1,2], Manuel Salto-Tellez[1, 2 *]

## Supplementary Figure Legends

### Supp Figure 1

The 3 distinctly heterogeneous areas are shown in columns A to C with their biomarker profiles beneath for p53, p-mTOR, EGFR and IGF1R.

### Supp Figure 2

The metastatic deposit of tumour in the lymph node is shown. Overall lymph node biomarker expression of p53, p-mTOR, EGFR and IGF1R are shown for each stain in column C. The interface between 2 heterogeneous metastatic areas is shown in column A. A third more dispersed, though morphologically distinct population is shown in column B.

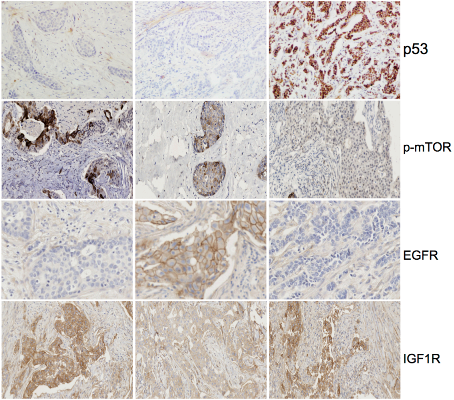
p53
p-mTOR
EGFR
IGF1R

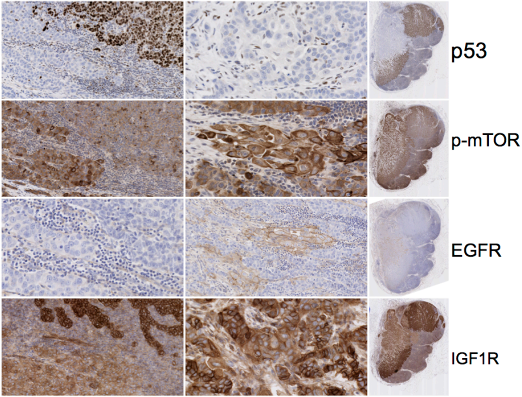
p53
p-mTOR
EGFR
IGF1R
